# Cost-effectiveness analysis of first and second-generation EGFR tyrosine kinase inhibitors as first line of treatment for patients with NSCLC harboring *EGFR* mutations

**DOI:** 10.1186/s12885-020-07329-8

**Published:** 2020-09-01

**Authors:** Oscar Arrieta, Rodrigo Catalán, Silvia Guzmán-Vazquez, Feliciano Barrón, Luis Lara-Mejía, Herman Soto-Molina, Maritza Ramos-Ramírez, Diana Flores-Estrada, Jaime de la Garza

**Affiliations:** 1 Thoracic Oncology Unit, Instituto Nacional de Cancerología, San Fernando No. 22, Col. Sección XVI, Del. Tlalpan, CP. 14080 Mexico City, Mexico; 2 HS Estudios Farmacoeconómicos, Mexico City, Mexico

**Keywords:** Lung adenocarcinoma, Treatment cost, Cost-effectiveness, Economic burden

## Abstract

**Background:**

Tyrosine-kinase inhibitors (TKIs) have become the cornerstone treatment of patients with non-small cell lung cancer that harbor oncogenic EGFR mutations. The counterpart of these drugs is the financial burden that they impose, which often creates a barrier for accessing treatment in developing countries. The aim if the present study was to compare the cost-effectiveness of three different first and second generation TKIs.

**Methods:**

We designed a retrospective cost-effectiveness analysis of three different TKIs (afatinib, erlotinib, and gefitinib) administered as first-line therapy for patients with NSCLC that harbor *EGFR* mutations.

**Results:**

We included 99 patients with the following TKI treatment; 40 treated with afatinib, 33 with gefitinib, and 26 with erlotinib. Median PFS was not significantly different between treatment groups; 15.4 months (95% CI 9.3–19.5) for afatinib; 9.0 months (95% CI 6.3- NA) for erlotinib; and 10.0 months (95% CI 7.46–14.6) for gefitinib. Overall survival was also similar between groups: 29.1 months (95% CI 25.4-NA) for afatinib; 27.1 months (95% CI 17.1- NA) for erlotinib; and 23.7 months (95% CI 18.6-NA) for gefitinib. There was a statistically significant difference between the mean TKIs costs; being afatinib the most expensive treatment. This difference was observed in the daily cost of treatment (*p <* 0.01), as well as the total cost of treatment (*p* = 0.00095). Cost-effectiveness analysis determined that afatinib was a better cost-effective option when compared with first-generation TKIs (erlotinib and gefitinib).

**Conclusion:**

In our population, erlotinib, afatinib, and gefitinib were statistically equally effective in terms of OS and PFS for the treatment of patients with advanced *EGFR*-mutated NSCLC population. Owing to its marginally increased PFS and OS, the cost-effectiveness analysis determined that afatinib was a slightly better cost-effective option when compared with first-generation TKIs (erlotinib and gefitinib).

## Background

Lung cancer is the leading cause of cancer-related deaths worldwide; among lung cancer types, the most frequently diagnosed is non-small cell lung cancer (NSCLC), which globally represents 85% of lung cancer cases. In 2018 GLOBOCAN reported an incidence for NSCLC of 7811 cases and an associated mortality of 6733 patients in Mexico, representing the sixth most commonly diagnosed neoplasia and third most common cause of cancer related-deaths [1]. At diagnosis, many patients with NSCLC are already in an advanced disease stage (IIIB or IV) and thus, they are ineligible for surgical resection. Therefore, the public health impact of lung cancer should be considered as a widespread problem that affects developed as well as developing countries [2]. Apart from the deleterious prognosis that lung cancer imposes, the financial burden that is associated with this neoplasia is overwhelming.

In the last decade treatment of patients with advanced NSCLC improved at an increased pace. The recognition of activating mutations and other biomarkers resulted in a paradigm shift for the treatment strategies of these tumors [3]. Currently, most patients with NSCLC receive a treatment scheme that includes targeted therapies such as EGFR tyrosine kinase inhibitors (TKI), immunotherapy, VEGF inhibitors or combination strategies [4]. These novel drugs have improved the prognosis of disease; however, their cost is significantly higher than the cost of previously used conventional chemotherapy.

Mutations of the gene *EGFR* are present on approximately 10–20% of patients with NSCLC, and in over 50% of patients with adenocarcinoma, which is the most frequent subtype among NSCLCs (45–55%) [5, 6]. TKIs, such as gefitinib, erlotinib, and afatinib, are the cornerstone drugs for the first-line treatment of patients with NSCLC harboring *EGFR* oncogenic mutations. Efficacy of first (gefitinib and erlotinib) and second-generation (afatinib) TKIs has been widely validated elsewhere [7–10]; it has been demonstrated that when used as first-line therapy in patients with advanced *EGFR*-mutated NSCLC the overall response rate (ORR) is ~ 70–80%, and the median progression-free survival (PFS) is 10–12 months [10, 11].

Recently, osimertinib a third-generation EGFR-TKI has demonstrated an overall survival benefit in the first-line setting against gefitinib or erlotinib, becoming the new standard of treatment in some developed countries [12, 13]. Nevertheless, osimertinib has not demonstrated to be cost-effective in most of the analyses conducted. Therefore, first-generation TKIs are still widely prescribed.

Even though the benefits of TKIs are enormous, the counterpart of these drugs is the financial burden that they impose. Additionally, TKIs are administered until disease progression or unacceptable toxicity, without any predetermined time of therapy, which further increases the cost of treatment [14]. The aim of this study was to retrospectively evaluate the cost-effectiveness of three different TKIs (afatinib, erlotinib, and gefitinib) in patients with *EGFR*-mutated NSCLC from a single tertiary-care medical center located at a developing country.

## Methods

### Study design

We developed a retrospective cost-effectiveness analysis of three different TKIs (afatinib, erlotinib, and gefitinib) administered as first-line therapy for patients with NSCLC that harbor *EGFR* mutations. For this analysis, we performed a Markov modeling with three possible patient health statuses: progression-free, progression of disease, or death.

### Data collection

Medical records from every patient with NSCLC and mutated *EGFR*, that received treatment at *Instituto Nacional de Cancerología* (INCan) at Mexico City, between January of 2013 and December of 2016, were reviewed. This was an observational study that did not jeopardized patients clinical management and or identity; therefore, approval by the ethics committee of INCan and signature of informed consent were both waived.

Analyzed data included: age, gender, Karnofsky performance status, ECOG performance status, biomass exposure, smoking history, diabetes mellitus, arterial hypertension, TKI therapy and adverse reactions to treatment (type, grade, duration, associated treatment and the treatment for adverse events). Data collection was performed between August of 2016 and June of 2017. Medical records from patients were excluded if the medical record was unable to report at least 80% of previously determined variables.

### Evaluation of monetary expenditure and cost-effectiveness analysis

Monetary expenditure estimation was developed by including the cost of corresponding TKI (afatinib, erlotinib or gefitinib); for this task, we considered the acquisition costs at which INCan purchased the drug (TKI). We also estimated the associated costs for treatment of unwanted effects that were related to each therapy; including medical visits and drugs used to palliate adverse effects, according to a preestablished INCan price list.

For the cost-effectiveness analysis, we calculated the Incremental Cost-Effectiveness Ratio (ICER), which is a summary measure representing the economic value of an intervention compared with an alternative. ICER was calculated with the following formula:

$$ \mathrm{ICER}=\left(\mathrm{Treatment}\ 1\hbox{-} \mathrm{Treatment}\ 2\right)/\left(\mathrm{Effectiveness}\ 1\hbox{-} \mathrm{Effectiveness}\ 2\right) $$

ICER reflects the cost per unit of effectiveness increased; if ICER stands below the acceptability threshold it can be assumed that the analyzed treatment is an appropriate cost-effectiveness option. For this study, the acceptability threshold was defined as the annual gross domestic product (GDP) per capita in Mexico; at the time of analysis, annual per capita GDP was 8902 USD. At a currency exchange of MXN 17.8 for each USD, the annual GDP was equivalent to MXN 158,455.00.

### Statistical analysis

For descriptive purposes, continuous variables were summarized as arithmetic means and standard deviations (SD), categorical variables were comprised as frequencies and proportions. Overall survival (OS), and progression-free survival (PFS) were analyzed by the Kaplan-Meier method. Statistical significance was predetermined to be present for values of *p* <  0.05 based on a two-sided test.

For deterministic sensitivity analysis, the time horizon was considered at 3 and 5 years, and the discount rate was determined according to the recommendations of the *Conduction of Economic Evaluation Studies of the National Health Council* guidelines. The deterministic sensitivity analysis was carried out considering the case-base of a 5% discount rate, also using 0, 3, and 7%, discount rates, as well as a probabilistic sensitivity analysis using Monte Carlo simulations. In total, 1000 simulation samples were randomly taken from the distributions, and each time, the model results (incremental costs and incremental effects) were recalculated.

All statistical analyses were carried out using the R software (version 3.6.2).

## Results

We included 99 patients with the following TKI treatment; 40 treated with afatinib, 33 with gefitinib, and 26 with erlotinib. The cost of TKIs, and treatment dosage used are shown in Table 1. Population baseline characteristics are presented in Table 2.

**Table 1:** Cost of Afatinib, Erlotinib, and Gefitinib; and indicated dosage

TKI	Presentation	Cost at which INCan bought one month of treatment	Daily dose
**Afatinib**	30 tablets of 40 mg	MXN 22,950.00	40 mg
**Erlotinib**	30 tablets of 150 mg	MXN 24,560.40	150 mg
**Gefitinib**	30 tablets of 250 mg	MXN 13,532.04	150 mg

**Table 2:** General characteristics of population

	Population ***N*** = 99	Afatinib ***N*** = 40	Erlotinib ***N*** = 26	Gefitinib ***N*** = 33
**Gender**				
***▪*** **Women**	73 (73.7%)	28 (70.0%)	22 (84.6%)	23 (69.7%)
***▪*** **Men**	26 (26.3%)	12 (30.0%)	4 (15.4%)	10 (30.3%)
**Age (years)**	61.2 (14.0)	57.925 (14.5)	64.3 (12.8)	62.8 (14.0)
**Stage IV NSCLC**	83 (83.8)	33 (82.5)	22 (84.61)	28 (84.84)
**Current or former smoker**	22 (22.2%)	3 (11.5%)	4 (12.1%)	15 (15.2%)
**Woodsmoke exposure**	16 (16.2%)	4 (15.4%)	6 (18.2%)	16 (16.2%)
**Karnofsky**				
• **50**	2 (2.2%)	1 (2.6%)	1 (4.2%)	0 (0.0%)
• **60**	3 (3.2%)	2 (5.3%)	0 (0.0%)	1 (3.2%)
• **70**	12 (12.9%)	4 (10.5%)	4 (16.7%)	4 (12.9%)
• **80**	33 (35.5%)	14 (36.8%)	7 (29.2%)	12 (38.7%)
• **90**	38 (40.9%)	16 (42.1%)	12 (50%)	10 (32.3%)
• **100**	5 (5.4%)	1 (2.6%)	0 (0.0%)	4 (12.9%)
• **Not Reported**	6	2	2	2
**ECOG**				
• **0**	2 (2.2%)	0 (0.0%)	0 (0.0%)	2 (6.5%)
• **1**	75 (81.5%)	34 (91.9%)	18 (75.0%)	23 (74.2%)
• **2**	12 (13.0%)	2 (5.4%)	4 (16.7%)	6 (19.4%)
• **3**	3 (3.3%)	1 (2.7%)	2 (8.3%)	0 (0.0%)
• **Not Reported**	7	3	2	2
**Diabetes Mellitus**	17 (17.2%)	5 (12.5%)	5 (19.2%)	7 (21.2%)
**Arterial hypertension**	23 (23.2%)	8 (20.0%)	7 (26.9%)	8 (24.2%)
***EGFR Mutation***				
***▪ EGFR del 19***	25 (25.3%)	12 (30.0%)	5 (19.2%)	8 (24.2%)
***▪ EGFR L858R***	67 (67.7%)	27 (67.5%)	16 (61.5%)	24 (72.7%)
***▪*** **Non-reported**	7 (7.1%	1 (2.5%)	5 (19.2%)	1 (3.0%)

**Fig. 1:**
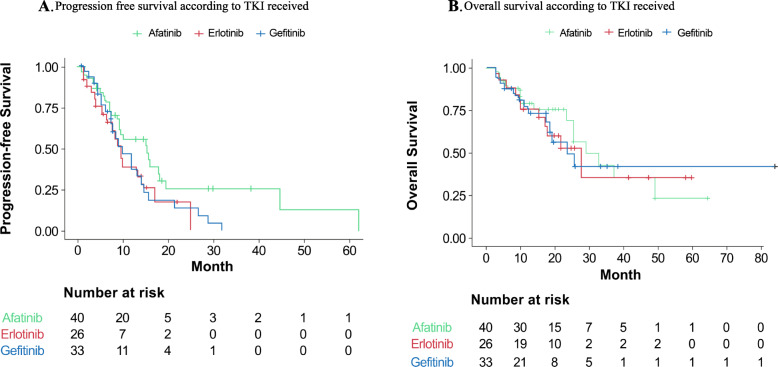
**a**. Progression free survival according to TKI received. **b**. Overall survival according to TKI received

Median PFS was not significantly different between treatment groups; 15.4 months (95% CI 9.3–19.5) for afatinib; 9.0 months (95% CI 6.3- NA) for erlotinib; and 10.0 months (95% CI 7.46–14.6) for gefitinib. Overall survival was also similar between groups: 29.1 months (95% CI 25.4-NA) for afatinib; 27.1 months (95% CI 17.1- NA) for erlotinib; and 23.7 months (95% CI 18.6-NA) for gefitinib. Kaplan Meyer curves of PFS and OS are presented in Fig. 1 a and b, respectively.

Adverse effects were present in 90% of patients treated with afatinib, 96.2% of patients treated with erlotinib, and 79% of patients treated with gefitinib. There were no statistically significant differences among adverse effects frequency between groups. Frequencies and grade of unwanted effects are presented at Table 3.

**Table 3:** Percentage of patients that developed adverse effects to TKI

Unwanted effect	Afatinib (n = 40)		Erlotinib (n = 26)		Gefitinib (n = 33)	
	Any Grade	Grade 3–4	Any Grade	Grade 3–4	Any Grade	Grade 3–4
Any unwanted effect	36 (90%)	3 (7.5%)	26 (100%)	4 (15.4%)	26 (78.8%)	1 (3%)
• Rash	23 (57.5%)	1 (2.5%)	13 (50%)	1 (3.8%)	13 (39.4%)	0 (0.0%)
• Diarrhea	14 (35%)	2 (5%)	13 (50%)	2 (7.7%)	18 (54.5%)	0 (0.0%)
• Xerosis	16 (40%)	0 (0.0%)	9 (34.6%)	0 (0.0%)	12 (36.4%)	0 (0.0%)
• Paronychia	11 (27.5%)	0 (0.0%)	4 (15.4%)	0 (0.0%)	4 (12.1%)	0 (0.0%)
• Nausea	3 (7.5%)	0 (0.0%)	7 (26.9%)	0 (0.0%)	7 (21.2%)	0 (0.0%)
• Acne	6 (15%)	0 (0.0%)	6 (23.1%)	0 (0.0%)	1 (3%)	0 (0.0%)
• Fatigue	5 (12.5%)	0 (0.0%)	6 (23.1%)	0 (0.0%)	2 (6.1%)	0 (0.0%)
• Stomatitis	6 (15%)	0 (0.0%)	5 (19.2%)	0 (0.0%)	5 (15.2%)	0 (0.0%)
• Constipation	2 (5%)	0 (0.0%)	4 (15.4%)	0 (0.0%)	3 (9.1%)	0 (0.0%)
• Vomit	2 (5%)	0 (0.0%)	3 (11.5%)	0 (0.0%)	1 (3%)	0 (0.0%)
• Alopecia	1 (2.5%)	0 (0.0%)	0 (0%)	0 (0.0%)	3 (9.1%)	1 (3%)
• Neuropathy	2 (5%)	0 (0.0%)	0 (0%)	0 (0.0%)	3 (9.1%)	0 (0.0%)
• Anorexia	3 (7.5%)	0 (0.0%)	0 (0%)	0 (0.0%)	1 (3%)	0 (0.0%)
• Liver toxicity	0 (0%)	0 (0.0%)	1 (3.8%)	0 (0.0%)	0 (0%)	0 (0.0%)
• Anemia	0 (0%)	0 (0.0%)	0 (0%)	0 (0.0%)	1 (3%)	0 (0.0%)
• Arthralgia	1 (2.5%)	0 (0.0%)	0 (0%)	0 (0.0%)	1 (3%)	0 (0.0%)

### Cost analysis results

Total and daily costs associated exclusively with each TKI is presented as mean, median, and range for each treatment group on Table 4. There was a statistically significant difference between the mean TKIs costs; being afatinib the most expensive treatment. This difference was observed in the daily cost of treatment (*p <* 0.01), as well as the total cost of treatment (*p* = 0.00095). The costs related to unwanted effects are also presented in Table 4. There were no statistically significant differences among total costs (medications plus medical visits) associated with unwanted effects between treatment groups. The total monetary cost of TKI plus unwanted effects was significantly higher in patients treated with afatinib.

**Table 4:** Cost of TKI therapy and cost of related unwanted effects (UE). Amounts are presented in MXN pesos

	Afatinib (*n* = 40)		Erlotinib (*n* = 26)		Gefitinib (*n* = 33)		*p*-value
	Mean (SD)	Median	Mean (SD)	Median	Mean (SD)	Median	
TKI cost							
Daily cost	722 (130)	765	794 (96)	819	451 (0)	451	< 0.001
Total cost	337,325 (299,832)	238,298	200,506 (137,831)	183,384	149,645 (107,139)	126,299	< 0.001
UE cost							
Medications	6846 (21,203)	2485	3479 (5727)	897	2212 (4762)	98	< 0.05
Consultation	4373 (5751)	1732	3797 (4382)	2598	4304 (6125)	1732	0.843
Total	11,219 (23,734)	6948	7276 (8498)	5267	6516 (8986)	2719	0.262
TOTAL TKI + UE	348,544 (311,386)	246,258	207,782 (143,566)	186,685	156,161 (111,330)	126,299	< 0.001

### Cost-effectiveness analysis

Results from the cost-effectiveness analysis are presented at Table 5.

**Table 5:** Cost-effectiveness analysis results according to PFS and OS for each treatment group. Amounts are presented in MXN pesos

TKI	Mean total cost	Incremental Cost	Effectiveness	Incremental Effectiveness	ICER
Progression Free Survival (PFS)					
Gefitinib	$161,800		8.18		Lowest cost
Erlotinib	$215,700	$53,900	6.7	−1.48	Dominated by gefitinib
Afatinib	$348,200	$186,400	9.46	1.28	$145,625.00 vs gefitinib
Overall Survival (OS)					
Gefitinib	$161,800		27.1		Lowest cost
Erlotinib	$215,700	$53,900	21.7	−5.4	Dominated by gefitinib
Afatinib	$348,200	$186,400	37.1	10	$18,640.00 vs gefitinib

As it can be seen, gefitinib has better effectiveness and lower cost than erlotinib; therefore, it can be stated that erlotinib is dominated by gefitinib in our cost-effectiveness analysis. Accordingly, any further analysis will only compare gefitinib and afatinib. While analyzing ICER between afatinib and gefitinib we can observe that afatinib is a better cost-effectiveness option when compared to gefitinib; this is because the obtained ICER is below the acceptability threshold, which was determined to be the annual GDP per capita in Mexico. By extended dominance, it could be assumed that afatinib is also a better cost-effective option than erlotinib.

### Deterministic sensitivity analysis

A deterministic sensitivity analysis was carried out upon the base case of a 5% discount rate, also using 0, 3, and 7% discounts. The results of this analysis for PFS are shown **in** Table 6.

**Table 6:** Deterministic sensitivity analysis results according to PFS. Amounts are presented in MXN pesos

TKI	Mean total cost	Incremental Cost	Effectiveness	Incremental Effectiveness	ICER
5% discount (case-base)					
Gefitinib	$161,800.00	–	8.18	–	–
Erlotinib	$215,700.00	$53,900.00	6.70	−1.48	Dominated
Afatinib	$348,200.00	$186,400.00	9.46	1.28	$145,625.00
No discount (0%)					
Gefitinib	$206,502.36	–	10.44	–	–
Erlotinib	$275,293.93	$68,791.58	8.55	−1.89	Dominated
Afatinib	$444,401.24	$237,898.88	12.07	1.63	$145,625.00
3% discount					
Gefitinib	$139,570.10	–	7.06	–	–
Erlotinib	$186,064.71	$46,494.61	5.78	−1.28	Dominated
Afatinib	$300,360.38	$160,790.28	8.16	1.10	$145,625.00
7% discount					
Gefitinib	$115,361.16	–	5.83	–	–
Erlotinib	$153,791.12	$38,429.96	4.78	−1.06	Dominated
Afatinib	$248,261.79	$132,900.62	6.74	0.91	$145,625.00

The univariate deterministic sensitivity analysis showed that for PFS, changing the discount rate does not modify the case-base results, reflecting robustness of the results from our analysis.

A probabilistic sensitivity analysis was also performed where a probabilistic distribution was assigned to health costs and results, considering a hypothetical cohort of 1000 patients via Monte-Carlo simulation; these results are presented at Table 7.

**Table 7:** Probabilistic sensitivity analysis results according to PFS and OS. Amounts are presented in MXN pesos

TKI	Mean total cost	Incremental Cost	Effectiveness	Incremental Effectiveness	ICER
Progression Free Survival (PFS)					
Gefitinib	$161,781.47	–	8.180	–	–
Erlotinib	$218,411.28	$56,629.81	6.701	−1.48	Dominated
Afatinib	$347,853.28	$129,442.00	9.457	1.28	$101,363.54
Overall Survival (OS)					
Gefitinib	$157,891.46	–	27.098	–	–
Erlotinib	$207,416.43	$49,524.98	21.774	−5.32	Dominated
Afatinib	$347,423.61	$140,007.18	37.095	10.00	$14,004.90

## Discussion

Treatment of patients with *EGFR*-mutated NSCLC has been revolutionized by TKIs, which are a clear example of the clinical benefit of target therapies. Albeit, the cost of these drugs is sometimes the greatest obstacle for obtaining this kind of treatment [14, 15]. In this retrospective study, we aimed to analyze the cost of therapy, the associated cost of treating unwanted effects related to therapy and the cost-effectiveness of three different TKIs; namely, erlotinib, gefitinib, and afatinib.

As our results suggest, all three studied TKIs are equally effective, with similar PFS and OS. We also noticed similar rates of unwanted effects in all the groups. These results are consistent with the meta-analysis published by Burotto et al, which reported similar effectiveness and security of afatinib, erlotinib, and gefitinib, while used as first-line therapy for patients with EGFR-mutated NSCLC [16]. Similarly, Liang et al. reported that erlotinib, afatinib, and gefitinib have similar effectiveness, however, they found increased toxicities in patients treated with erlotinib and afatinib, especially rash and diarrhea [17].

It is important to underscore that none of the prior mentioned studies was a randomized controlled clinical trial, therefore conclusions should be made cautiously while analyzing their results. The LUX-Lung 7 was the first randomized clinical trial that compared two different TKIs, afatinib versus gefitinib, as first-line therapy in patients with NSCLC with *EGFR* gene mutations. In this trial, afatinib provided a marginally benefit in PFS and time to a treatment failure when compared with gefitinib; these results demonstrated to be constant in every subgroup, including patients with L858R mutations and those with deletions on exon19. However, differences in the median OS for both arms were not significantly different, and afatinib present more grade ≥ 3 adverse events and serious adverse events. In our study, we did not detect significant differences in PFS or OS among the three treatment groups; however, afatinib was associated with the longest median PFS and OS [18].

The cost-effectiveness analysis of frequently prescribed drugs is becoming of great value for oncologists and decision-makers, especially for the new and upcoming drugs [19, 20]. Thus, cost-effectiveness analyses must consider costs of adverse events management, traveling, and productivity losses, besides the acquisition costs. In a European study, afatinib had the highest probability of being cost-effective (43%) compared to other TKIs (erlotinib, gefitinib, and osimertinib). Meanwhile, the probability of being cost-effective for gefitinib, erlotinib, and osimertinib was 13, 19, and 26%, respectively, at the Dutch threshold of €80,000/QALY [21]. In the present study, the cost-effectiveness analysis determined that afatinib was a better cost-effective option when compared with erlotinib and gefitinib at a Mexican threshold of MXN 158,455.

The limitations of our study are the retrospective nature of the design, which might misreport the complete expenditures associated with cancer treatment; besides, being performed at a single healthcare facility from a developing country with a relatively weak economy, the population might significantly differ from populations at first-world countries with more developed healthcare systems, in which many patients count with private healthcare insurance.

To the best of our knowledge, this was the first study in the Latin-American population that compared the cost-effectiveness of first-line treatment TKI’s (gefitinib, erlotinib, and afatinib) for *EGFR*-mutated (exon 19 deletion or exon 21 L858R mutation) NSCLC patients. Furthermore, in our study, we were able to obtain enough statistical power to determine that even if afatinib is the most expensive treatment, the increased monetary expenditure is compensated with increased effectiveness, although this increased effectiveness did not reach statistical significance. These results prevailed at the deterministic and probabilistic sensitivity analysis; therefore, our results should be considered robust.

Regarding osimertinib as an option of treatment, many cost-effective analyses have been performed to determine its cost-effectiveness compared to first and second-generation TKIs in the first-line, and after progression to previous TKI treatment in patients harboring a T-790 M resistance *EGFR* mutation; none of the aforementioned studies have demonstrated that currently osimertinib is a better cost-effective option of treatment [22–24]. Additionally, those studies were developed in financially stronger health-care systems than ours. It should be noted that most of the population in Mexico has an economical access barrier for acquiring osimertinib, which renders first and second generation TKIs as the most frequently used drugs to treat patients with *EGFR*-mutated NSCLC in our country.

## Conclusions

Cost-effectiveness analyses are gaining tremendous relevance, especially while treating patients with limited monetary resources, such as patients treated at our cohort. In our population, erlotinib, afatinib, and gefitinib were equally effective in terms of OS and PFS for the treatment of patients with advanced *EGFR*-mutated NSCLC population. Moreover, adverse events were not significantly different, rendering their security profiles quite similar. Afatinib was the most expensive drug, however, owing to its increased PFS and OS, the cost-effectiveness analysis determined that, in the setting of a developing country with a weak economy, afatinib was a slightly better cost-effective option when compared with first-generation TKIs (erlotinib and gefitinib).

## Data Availability

All data generated and analyzed during this study are included in this manuscript. Datasets are available through the corresponding author on reasonable request.

## Abbreviations

EGFR: Epidermal Growth Factor Receptor
TKI: Tyrosine kinase Inhibitor
NSCLC: Non-small Cell Lung Cancer
VEGF: Vascular-endothelial Growth Factor
ORR: Overall Response Rate
PFS: Progression Free Survival
OS: Overall Survival
ICER: Incremental Cost-Effectiveness Ratio
INCan: *Insituto Nacional de Cancerología*
